# Keeping track of who said what: Performance on a modified auditory *n*-back task with young and older adults

**DOI:** 10.3389/fpsyg.2015.00987

**Published:** 2015-07-22

**Authors:** Gary R. Kidd, Larry E. Humes

**Affiliations:** Department of Speech and Hearing Sciences, Indiana UniversityBloomington, IN, USA

**Keywords:** hearing, speech perception, effort, working memory, aging

## Abstract

A modified auditory *n*-back task was used to examine the ability of young and older listeners to remember the content of spoken messages presented from different locations. The messages were sentences from the Coordinative Response Measure (CRM) corpus, and the task was to judge whether a target word on the current trial was the same as in the most recent presentation from the same location (left, center, or right). The number of trials between comparison items (the number back) was varied while keeping the number of items to be held in memory (the number of locations) constant. Three levels of stimulus uncertainty were evaluated. Low- and high-uncertainty conditions were created by holding the talker (voice) and nontarget words constant, or varying them unpredictably across trials. In a medium-uncertainty condition, each location was associated with a specific talker, thus increasing predictability and ecological validity. Older listeners performed slightly worse than younger listeners, but there was no significant difference in response times (RT) for the two groups. An effect of the number back (*n*) was seen for both PC and RT; PC decreased steadily with *n*, while RT was fairly constant after a significant increase from *n* = 1 to *n* = 2. Apart from the lower PC for the older group, there was no effect involving age for either PC or RT. There was an effect of target word location (faster RTs with a late-occurring target) and an effect of uncertainty (faster RTs with a constant talker-location mapping, relative to the high-uncertainty condition). A similar pattern of performance was observed with a group of elderly hearing-impaired listeners (with and without shaping to ensure audibility), but RTs were substantially slower and the effect of uncertainty was absent. Apart from the observed overall slowing of RTs, these results provide little evidence for an effect of age-related changes in cognitive abilities on this task.

## Introduction

Many people have difficulty participating in conversations when listening conditions are not ideal. Speaking with one person face-to-face in a quiet environment is considerably easier than conversing with a group in a noisy restaurant, and the difficulty tends to be greater for older listeners, especially those with hearing loss (Humes, 2002; Humes and Dubno, 2010). Many factors can make listening conditions more difficult, including background noise, competing speech sounds, reverberation, poor enunciation, and other types of distraction or signal degradation (see Mattys et al., 2012, for a recent review). One very common factor, often confounded with background noise, is the presence of multiple talkers participating in a group conversation. Such conversations often take place in noisy environments with the participants talking over each other; but even in a quiet environment with polite turn taking, this can be a challenging listening situation for some listeners. Following a sequential or turn-taking multitalker conversation generally requires a listener to keep track of interleaved remarks from multiple talkers. Listening to each new contribution to a conversation while remembering earlier remarks from other talkers and correctly attributing those remarks to the different participants places demands on cognitive abilities that tend to decline with age.

Many studies have shown that cognitive abilities generally diminish with age (see Salthouse, 2010, 2012, for reviews), but the degree to which this reduces the ability to follow a multitalker conversation is not clear. The task of following a conversation consisting of interleaved messages from multiple talkers may involve several distinct abilities. Although a multitalker conversation puts demands on working memory, in that listeners are required to keep information in memory while processing new information from each new talker, it also involves other abilities that may be independent of the simultaneous memory and processing abilities assessed by working memory tasks. For example, localization ability, selective listening abilities, the ability to make use of partial information, and the ability to deal with uncertainty may all come into play in a multitalker listening situation. These abilities may be largely independent and may not be affected by aging in the same way.

The implications of age-related changes in cognitive abilities for speech understanding are not always clear. When present, hearing loss is often the primary reason for a decrease in speech-understanding ability with increasing age, but cognitive factors also play an important role. The role of cognitive factors is most apparent when speech is presented in a background of competing speech or speech-like sounds and when the speech is amplified to ensure audibility (see Akeroyd, 2008; Houtgast and Festen, 2008; Humes and Dubno, 2010; Humes et al., 2013). There are very large individual differences in speech-understanding abilities at all ages, so one must be careful about generalizations concerning the abilities of younger vs. older listeners. Even with audible (amplified) speech, older listeners often perform worse than younger listeners under difficult listening conditions (e.g., Humes et al., 2006; Humes and Coughlin, 2009; Kidd and Humes, 2012). However, with fully audible speech, older subjects also perform as well as younger listeners on many difficult speech-understanding tasks (e.g., Humes et al., 2013). Moreover, with highly predictable speech stimuli that provide linguistic and prosodic context, older listeners often outperform younger listeners (e.g., Pichora-Fuller et al., 1995; Wingfield et al., 2000; Humes et al., 2013).

Given the large individual differences in speech-understanding abilities and the dependency of age effects on the specifics of the speech task, it is difficult to predict how age-related changes in hearing and cognition will affect performance in more complex everyday listening situations. Much of what is known about the influence of hearing loss and cognitive abilities on speech understanding comes from studies that require subjects to recall words immediately after presentation of a single word or sentence. However, in everyday listening situations, successful communication requires more than recognition and immediate recall. Although some researchers have examined age differences in the performance of more complex speech-understanding tasks (e.g., Pichora-Fuller et al., 1995; Schurman et al., 2014), much remains unknown about how older listeners are affected by the increased cognitive demands of real-world conversational tasks.

The present study uses an approximation of a multitalker sequential conversation to examine the influence of several factors on the ability to understand and recall information in a series of spoken sentences. To assess the role of aging and hearing loss on this task, the study employs young, normal-hearing (YNH) adults, and older adults, with and without hearing loss. A modified auditory *n*-back task was used with spoken sentences as stimuli. This paradigm, described in more detail below, provides a means of assessing memory for words in different sentence positions under different levels and types of uncertainty, or variability, across trials. The *n*-back task provides a convenient framework for examining these variables in an experimental paradigm that has many features in common with a sequential or turn-taking multitalker conversation.

### The *n*-back task

The *n*-back task is widely used as a measure of working memory, especially in cognitive neuroscience research (e.g., Cohen et al., 1997; Owen et al., 2005). The task requires subjects to judge whether information presented on the current trial matches that presented on an earlier trial, one or more (*n*) trials back in a sequence of trials. To perform this task, a subject must hold the last *n* items in memory, so that the identity of the item *n* presentations prior to the current one is always available as new items are presented. For this basic version of the task, *n* is therefore equal to both the number of presentations back in the sequence for the comparison item and the memory set size. The task is typically performed in the visual modality with single letters or digits presented individually in a sequence. Many variants of this task (including different presentation strategies, stimuli, and presentation modalities) have been used to test various hypotheses concerning control processes and memory systems in working memory (e.g., McElree, 2001; Oberauer and Bialkova, 2009; Basak and Verhaeghen, 2011; see Owen et al., 2005; Redick and Lindsey, 2013, for reviews). Like any working memory task, the *n*-back task has some task-specific demands that involve abilities that may have little or nothing to do with the basic processing and capacity aspects of working memory (see Kane et al., 2007; Schmiedek et al., 2009). Moreover, *n*-back tasks have been shown to have a fairly weak correlation with other measures of working memory that consist of interleaved memory and processing tasks (Redick and Lindsey, 2013). These “complex-span” tasks (e.g., reading span, operation span; see Conway et al., 2005, for a summary) have been more popular than *n*-back tasks as measures of working memory in most research on individual differences in cognition (e.g., Daneman and Merikle, 1996; Unsworth and Engle, 2007). The substantial differences in performance on various working memory tasks show that working memory is a complex construct that cannot be effectively assessed with a single measure. However, the *n*-back task has some properties that make it useful for the assessment of certain features of working memory in the context of a sequential multitalker conversation.

In the present study, a modified *n*-back task is used to measure the ability to recall information in sentences spoken by different talkers at different times in a series of spoken messages. The use of this type of task makes it possible to assess components of working memory (such as focus switching and memory for items outside the focus of attention) and determine their influence on the ability to follow a multitalker conversation. The modification of the *n*-back task used here is similar to that used by Verhaeghen and Basak (e.g., Verhaeghen and Basak, 2005; Basak and Verhaeghen, 2011) and Oberauer (2002, 2006) in their investigations of working-memory processes with a visual *n*-back task. Their work has examined effects of aging on the ability to switch items stored in memory in and out of the focus of attention (focus switching) and the probability of recalling items stored outside of the focus of attention (item availability). The research is guided by a two-stage model of working memory (see Cowan, 2001) that posits two memory stores in working memory: a very limited capacity store that affords immediate access (i.e., the focus of attention) and a larger “outer” store in which items are in an activated or available state, but not accessible until they are brought into the focus of attention (with a “focus switch”). With the *n*-back task, focus switching and item search time (for items in the outer store) can be assessed by measuring the time required to judge whether the current item was repeated *n* trials ago as a function of *n* (which is also equal to the number of items that must be held in memory to perform the task). Assuming a 1-item capacity for focus of attention, response times (RT) for *n* = 1 trials can be compared to that for *n* = 2 trials to obtain a measure of switching time. This is because on one-back trials, the current item is compared to the immediately preceding item, an item that is still held in the focus of attention, while on two-back trials, an item must be switched from the outer store into the focus of attention. Any increase in RT with further increases in *n* indicates search time for items in the outer store. In addition to memory search efficiency, the *availability* of items in the outer store can be assessed by examining percent-correct performance as a function of *n*.

The standard version of the *n*-back task has two characteristics that make it unsuitable for assessing sequential multitalker-conversation abilities. First, in conversation we need to keep track of who said what, but a precise ordering of the different participants' contributions to the conversation is generally not important, as long as we follow the flow of the conversation. That is, we can generally follow a conversation quite well even if we are not sure whether two or three other people have spoken since the person sitting next to us last spoke. Second, people do not contribute to a conversation in a fixed order, with everyone contributing once before contributing again. Both of these constraints can be eliminated with an auditory *n*-back task simply by presenting stimuli from fixed locations and asking subjects to judge whether the stimulus they just heard in a given location is the same as the last one they heard in that same location. With this task, the number of items to be remembered (or set size) is equal to the number of locations used. Further, if stimuli are presented from different locations in an unpredictable order, the number of trials between the current stimulus and the comparison stimulus (i.e., the number back, *n*) can be varied independently of the set size and can even exceed the set size.

This type of auditory *n*-back task is illustrated in Table 1 using a set size of 3 (i.e., three locations). As illustrated, the subject must retain both what was said (a spoken digit in this example) and from where it originated (left, center, or right). With three locations, the subject must remember only three digits and simply indicate “yes” or “no” to indicate whether the digit just heard matches the last digit heard from the same location. As noted, the accuracy of the responses is recorded together with the RTs and both are examined as a function of *n*.

**Table 1:** **An example of 10 trials of an auditory ***n***-back task with spoken digits presented from three locations; left, center, or right**.

**Trial**	**LEFT**	**CENTER**	**RIGHT**	**Trial type**
1	“two”			No
2		“six”		No
3			“seven”	No
4		“six”		*n* = 2, Yes
5	“three”			*n* = 4, No
6	“three”			*n* = 1, Yes
7		“five”		*n* = 3, No
8		“eight”		*n* = 1, No
9			“seven”	*n* = 6, Yes
10	“four”			*n* = 4, No

*The subjects task is simply to indicate (yes or no) whether the digit just heard is the same as the previous digit heard from that same location. The value of n is the number of trials back in the sequence for the comparison*.

Verhaeghen and Basak (Verhaeghen and Basak, 2005; Basak and Verhaeghen, 2011) have used a visual *n*-back task that shares some properties with the present task. For a set size of one, when focus switching is never required, older adults were as accurate as younger adults and performed nearly perfectly. However, for set sizes greater than one, older adults were less accurate than younger ones on one-back trials (not requiring a focus switch) as well as on trials that did require a focus switch (i.e., trials with a comparison more than one back in the series of presentations). Thus, the burden of keeping track of more than one location for target numbers (and/or maintaining one or more items in the outer store) had a negative effect on older adults' performance, even on trials that did not require focus switching. This shows that, at least under some conditions, older adults have more difficulty maintaining information both inside and outside the focus of attention than do younger adults. However, no differences were found between young and older subjects in focus-switching costs, measured by response times, when general slowing was taken into account. That is, the relative increase in RTs between one-back trials and two-back (or greater) trials was approximately the same for younger and older subjects.

The present studies provide measures of these working-memory processes (i.e., focus switching, memory search, and availability of information outside focal attention) in the context of an auditory *n*-back task that has some of the properties of a sequential multitalker conversation. Similar to the illustration in Table 1, full sentences are presented auditorily from different apparent locations (left, center, or right) over headphones. Subjects are asked to judge whether a target word in the sentence they just heard is the same as that in the most recent sentence presented from the same location. This creates a more natural task that resembles the task of listening to three people (separated in space) and keeping track of who said what.

Although subjects are asked to remember (and compare) only one key word in each sentence, the additional information in the full sentence adds to the processing burden and is potentially distracting. Moreover, the use of apparent location to indicate the stimulus to be compared to the current stimulus may not be as effective as column position in a visual display, as used by Verhaeghen and Basak (2005), because of both age-related changes in localization ability (see Dobreva et al., 2011) and differences between memory for auditorily specified location and memory for location in a two-dimensional visual array (see Parmentier and Jones, 2000; Martin et al., 2011).

In addition to the changes in modality and stimulus complexity, the current studies also differ from earlier *n*-back studies by including a manipulation of the variability in the sentences across trials as a way to measure effects of complexity and stimulus uncertainty on performance. This includes a condition in which the same sentence spoken by the same talker is used across trials with only a change in the key word (minimum uncertainty), plus a condition with variation in talkers and sentences across trials (maximum uncertainty). A third condition more closely approximates a real sequential multitalker situation by having a constant talker-location correspondence while maintaining the same stimulus variability as the maximum uncertainty condition. This medium-uncertainty condition provides a test of the potential benefit due to the ecological validity of each location being associated with a different specific voice (or person) as well as the potential benefit due to comparisons of words spoken in the same voice.

Although the modified *n*-back task used in this study does not have all of the characteristics of a real sequential multitalker conversation, the task and the various conditions used allow for tests of the role of many factors that may play an important role in the ability to follow a real-world multitalker conversation. These include focus switching, memory search, the availability of items in memory (outer store), cognitive load, distraction, uncertainty, the use of location cues, and the use of indexical properties of speech. To determine how these factors are affected by hearing loss and aging, two experiments were conducted: one with young and older adults with normal hearing, and one with older hearing-impaired adults tested with and without spectral shaping (amplification) to ensure full audibility of the stimuli.

## Experiment 1: Young and older adults with normal hearing

The first experiment examined performance on a modified auditory *n*-back task by younger and older adults with normal hearing. Based on performance with a similar visual task (see Oberauer, 2006; Basak and Verhaeghen, 2011), it was expected that older subjects would be slower and less accurate than younger subjects, but that the two groups would have similar switching costs, as evidenced by the relative increase in RT from *n* = 1 (when no focus switching is required) to *n* = 2 (when focus switching is required). The increased processing load due to the use of full sentences, rather than single letters or numerals, was expected to have a greater impact on the older listeners. This would lead to larger age differences in percent-correct performance than seen in related earlier studies with simpler stimuli, and possibly to reduced efficiency in memory search, which would tend to increase RTs on trials with *n* > 1, due to slower searching for items in the outer store. The use of target words early and late in the sentence provides a test of potential memory interference due to irrelevant information preceding or following the target word. Finally, the use of the different uncertainty conditions provides a test of the effect of stimulus variability on younger and older listeners as well as a test of the possible benefit due to the consistent mapping of voices to locations, which more closely approximates an everyday listening situation.

### Methods

#### Subjects

Two groups of listeners participated in Experiment 1. The young, normal-hearing (YNH) group consisted of 10 young adults (3 men and 7 women) between the ages of 20 and 24 years (mean = 22.2 years; *SD* = 1.3). The older normal-hearing (ONH) group consisted of 12 older adults (6 men and 6 women) between the ages of 61 and 72 years (mean = 66.2 years; *SD* = 3.5). All YNH listeners had pure tone thresholds ≤ 25dB HL (ANSI, 2004) for all octave frequencies between 250 and 8000 Hz. ONH listeners were required to have a pure tone average (PTA_500,1000,2000Hz_) ≤ 15 dB HL and a high-frequency PTA (HFPTA_1000,2000,4000Hz_) ≤ 25 dB HL. All subjects had normal tympanograms and otoscopic findings and showed no evidence of middle ear pathology. The YNH subjects were students at Indiana University in Bloomington and the ONH subjects were from the Bloomington, Indiana community. The ONH subjects (with 2 exceptions) had served in an earlier individual differences study (Humes et al., 2013), which had included screening for serious cognitive and physical impairment. The highest level of education completed ranged from high school (one subject) to vocational school (two subjects), college (five subjects), and graduate school (four subjects). All subjects were native speakers of English and were paid for their participation. Subject recruitment and all experimental procedures were reviewed and approved by the IRB at Indiana University.

#### Stimuli

The stimuli were sentences from the Coordinate Response Measure (CRM) Corpus (Bolia et al., 2000). This corpus consists of a collection of sentences spoken by four male and four female talkers. All sentences are of the form “Ready [call sign] go to [color] [number] now.” There are eight call signs (arrow, baron, charlie, eagle, hopper, laker, ringo, tiger), four colors (blue, green, red, white), and eight numbers (1–8) spoken in all 256 combinations by each talker. Three talkers (two male and one female), judged to be maximally distinguishable by three research assistants, were selected for this study.

Stimuli were presented at 85 dB SPL. This relatively high presentation level was used to approximate the levels used with the older hearing-impaired (OHI) listeners in Experiment 2. For those listeners, the stimuli were amplified to ensure audibility (at least 13 dB above threshold) for frequencies from 125 to 4000 Hz, often resulting in presentation levels above 80 dB SPL. Previous work has shown that presentation levels in this range generally lead to slightly poorer intelligibility (e.g., Dubno et al., 2005a,b; Studebaker et al., 1999) in normal-hearing listeners.

#### Procedures

All testing was done in a sound-treated booth that met or exceeded ANSI guidelines for permissible ambient noise for earphone testing (American National Standards Institute, 1999). Stimuli were presented binaurally, using Etymotic Research ER-3A insert earphones. Stimuli were presented by computer using a Digital Audio Labs Card Deluxe sound card and a Tucker Davis Technologies System-3 HB7 headphone buffer. Each listener was seated in front of a touchscreen monitor, with a keyboard and mouse available.

On each trial, a single CRM sentence was presented to the left, right, or both earphones to simulate left, right, or center locations, respectively, for the apparent source. All subjects reported that the three apparent source locations were easily identified. Each trial began with the word “LISTEN” presented visually on the display, followed 500 ms later by presentation of a sentence. After each presentation, subjects responded by touching (or clicking with a mouse) one of two virtual buttons (labeled “yes” and “no”) on a touch screen display to indicate whether the target word (either the number or the call sign) was the same as that spoken by the last talker heard from the same location. The next trial was presented immediately after the subject responded. No feedback was provided (except during practice trials, described below). Subjects were told to respond as quickly as possible without making errors and were encouraged to guess when they felt unsure of the correct response.

A trial block consisted of a sequence of 33 trials with location repetitions beginning on the fourth trial. An example of the first 10 trials of a block is shown in Table 2, with number as the target word. The first three trials were always presented in the left, center, and right virtual locations, in that order, and subjects were instructed to respond “no” to those trials (the “yes” option did not appear) since there was no repetition of any location. This resulted in 30 observations per trial block. The contents of each of the 33 trials in a block were randomized with the following constraints. Within the sequence of 33 trials, each virtual location was used 11 times. The number of trials since the last presentation in a given location (*n*) ranged from 1 to 5, with 6 repetitions of each value of *n* in each block of trials. Each of the 8 target words (call signs or numbers) was used at least twice and no more than 8 times within a trial block. All subjects began with four practice trial blocks: two with the number target, followed by two with the call sign target. During the practice trials, correct/incorrect feedback was provided on every trial.

**Table 2:** **An example of 10 trials of the modified auditory ***n***-back task, with number as the target word**.

**Trial**	**LEFT**	**CENTER**	**RIGHT**	**Trial Type**
1	Ready Baron go to blue TWO now			No
2		Ready Ringo go to red SIX now		No
3			Ready Hopper go to white SEVEN now	No
4		Ready Baron go to blue SIX now		*n* = 2, Yes
5	Ready Eagle go to green THREE now			*n* = 4, No
6	Ready Charlie go to white THREE now			*n* = 1, Yes
7		Ready Laker go to green FIVE now		*n* = 3, No
8		Ready Tiger go to red EIGHT now		*n* = 1, No
9			Ready Tiger go to green SEVEN now	*n* = 6, Yes
10	Ready Arrow go to blue FOUR now			*n* = 4, No

*This example shows the variable nontarget and color words used in the medium and high uncertainty conditions. In the medium-uncertainty condition, each of three talkers is associated with one of the three locations. In the high-uncertainty condition, the locations of the three talkers vary randomly across trials. A single talker is used with the same call sign and color on every trial in the minimal-uncertainty condition*.

In three different uncertainty conditions, the selection of non-target words in the sentences and the assignment of talkers (voices) to different locations were varied. (See Table 2 for an example of nontarget word variation.) In the low uncertainty condition, the same voice was used on every trial (the same male voice for all subjects) and all words in the sentence other than the target word (call sign or number) were the same on every trial. In the high uncertainty condition, the talker and the two variable nontarget words (color and either call sign or number) were selected randomly on each trial. A third, more ecologically valid, condition had the same random variation in nontarget words, but had a consistent mapping of talker and location. This medium uncertainty condition creates the impression of a different specific person at each location, while maintaining the same amount of stimulus variability as in the high uncertainty condition. The factorial combination of these three conditions with the two Target conditions (call sign and number) resulted in six conditions. There were eight trial blocks in each condition, for a total of 30 × 8 = 240 observations per condition. Subjects were not told about the differences across conditions in the number of talkers, sentence variability, or the assignment of talkers to locations.

All subjects were presented with all six conditions, with a different counterbalanced order of conditions for each subject. Trial blocks were run in sets of four with no experimenter intervention between trial blocks within a set. All trial blocks within a set were in the same Target condition. The experimenter announced the identity of the target word at the beginning of each set and a reminder of the current target (“call sign” or “number”) was displayed at the top of the screen throughout each trial block. The Target condition changed with each successive set, and the Uncertainty condition was held constant for two consecutive sets (one in each of the two Target conditions). Each counterbalanced order was created by using one of six possible orders of the three Uncertainty conditions and alternating Target conditions within each Uncertainty condition, starting with either call sign or number as target. One set of four trial blocks in each condition was run in the first test session, followed by a second set of four trial blocks in each condition in the second session, using the same order of conditions in each session. Testing was completed in two 90-min sessions on separate days.

### Results

Response time (RT) and response correctness were scored on each trial. Response time was measured from the appearance of the “Yes” and “No” virtual buttons on the screen to the mouse click (or touch) on a button. Only RTs for correct responses were used in the analysis. Extreme fast and slow responses were omitted by excluding all RTs less than 150 ms and all RTs greater than three times the standard deviation above the mean for each condition. Using these exclusion criteria, the average number of excluded responses across conditions was less than three percent (almost entirely due to slow responses). For the purposes of statistical analysis, the percent-correct (PC) scores were converted to rationalized arcsine units (RAU; Studebaker, 1985).

Overall, performance was very good, with PC scores ranging from 80 to 96% (mean = 89%, *SD* = 5.4%) for the YNH listeners and from 62 to 94% (mean = 78%, *SD* = 9.3%) for the ONH listeners. Response times were similar to those found for other versions of the *n*-back task for the younger listeners (mean *RT* = 780 ms, *SD* = 295 ms), but RTs for the older listeners (mean = 893 ms, *SD* = 258 ms) were more similar to the younger listeners than typically observed (see Verhaeghen and Basak, 2005; Basak and Verhaeghen, 2011).

**Figure 1:**
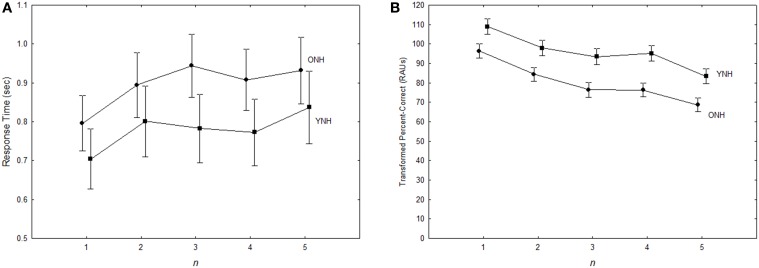
**Response times for correct responses (A) and accuracy (B) as a function of the number of presentations separating the target words to be compared, for both age groups in Experiment 1**. Error bars indicate ± one standard error.

The main results are summarized in Figure 1. Performance is shown as a function of *n* for both groups, with RT shown in Figure 1A and transformed percent correct (tPC) in RAU shown in Figure 1B. (Recall that *n* is the number back and the set size is constant at 3.) A 2 (Group) × 3 (Uncertainty) × 2 (Target) × 5 (*n* back) analysis of variance performed for both tPC and RT revealed that the group difference in RT was not significant (*F* < 1.0), while the difference in accuracy was significant [*F*_(1, 20)_ = 9.84; *p* < 0.01, $\eta_{\text{p}}^{2}=0.33$]. There were no interactions with group in either analysis (*p* > 0.05). Thus, both younger and older listeners with normal hearing were found to be affected by the experimental manipulations in the same way, with younger listeners significantly outperforming the older ones only in terms of accuracy. Because there were no interactions with the group variable, discussion of the effects of the within-group variables are presented below without a separate analysis for each group, although group-specific data will continue to be depicted descriptively in subsequent figures.

#### Performance as a function of *n*

It can be seen in Figure 1 that the effect of *n* was quite similar for the two groups for both RT and accuracy: RT tends to rise and accuracy tends to fall as *n* increases. Analysis of RTs revealed a significant main effect of *n* [*F*_(4, 80)_ = 11.1, *p* < 0.001,$\eta_{\text{p}}^{2}=0.36$]. However, follow-up analyses revealed that only the difference between *n* = 1 and *n* = 2 was significant (Tukey HSD; *p* < 0.001), with no significant differences for any further increases (*p* > 0.25). This indicates that both groups have the same cost (about 100 ms) for switching information in and out of working memory, and the same efficiency of memory search for items in the outer store. A significant *n* x Uncertainty interaction [*F*_(8, 160)_ = 2.9, *p* < 0.01, $\eta_{\text{p}}^{2}=0.13$] reflected a slight flattening of the RT function with an increase in uncertainty, with a significant difference between *n* = 1 and *n* = 2 only in the low-uncertainty condition (Tukey HSD, *p* < 0.001). This suggests that increased complexity of full sentences and irrelevant stimulus variability make it more difficult to access the most recent target word in memory. A significant three-way interaction [*F*_(8, 160)_ = 2.3, *p* < 0.05, $\eta_{\text{p}}^{2}=0.10$] reflected a larger performance decrement in the high-uncertainty condition with the call-sign target, especially for the lower values of *n*.

There was also a significant main effect of *n* for tPC scores [*F*_(4, 80)_ = 61.3, *p* < 0.001, $\eta_{\text{p}}^{2}=0.75$], with a negative accuracy slope of approximately 4 RAU. Each increase in *n* resulted in a significant decrease in tPC (Tukey HSD, *p* < 0.05), except for the difference between *n* = 3 and *n* = 4 (*p* > 0.9). The fairly constant difference between the two groups at all values of *n* shows that an increase in the time (and number of intervening items) between items to be compared resulted in similar decreases in the availability of items for young and older listeners.

Significant two-way interactions reflected slight differences in the rate of decrease in accuracy with increases in *n* in the different conditions. A significant Target x *n* interaction [*F*_(4, 80)_ = 4.1, *p* < 0.005, $\eta_{\text{p}}^{2}=0.17$] was associated with a substantially greater difference between performance for *n* = 4 and *n* = 5 for the call sign target than for the number target, and a significant Uncertainty x *n* interaction [*F*_(8, 160)_ = 3.7, *p* < 0.001, $\eta_{\text{p}}^{2}=0.16$] was due to a considerably smaller difference between *n* = 1 and *n* = 2 in the high uncertainty condition than in the other uncertainty conditions. Finally, a significant three-way interaction was primarily due to the latter two-way interaction being greater for the call sign target than for the number target.

#### Performance under different levels of uncertainty

**Figure 2:**
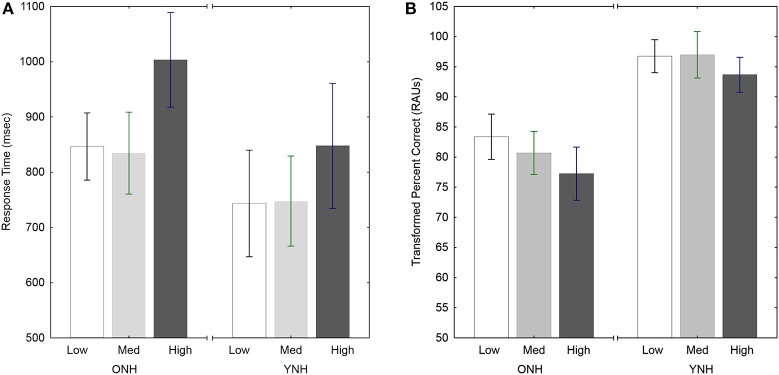
**Response times (A) and accuracy (B) for both groups of subjects in the three Uncertainty conditions of Experiment 1**. Error bars indicate ± one standard error.

Figure 2 shows the effect of uncertainty for YNH and ONH subjects for both RT and accuracy. Although performance was worst in the high-uncertainty condition for both measures, the pattern was slightly different for RT and tPC. Both main effects of uncertainty were significant [RT: *F*_(2, 40)_ = 12.3, *p* < 0.001, $\eta_{\text{p}}^{2}=0.38$; tPC: *F*_(2, 40)_ = 4.5, *p* < 0.05, $\eta_{\text{p}}^{2}=0.18$] and follow-up tests (Tukey HSD) indicated a similar pattern of significance for both RT and tPC. For RT, the high-uncertainty condition was significantly slower than the other uncertainty conditions (*p* < 0.001), which were not different from each other (*p* > 0.9). For tPC, the low- and medium-uncertainty conditions were not significantly different (*p* > 0.6), and the high-uncertainty condition was significantly more difficult than the low-uncertainty condition (*p* < 0.05). However, the difference between the high- and medium-uncertainty conditions was only marginally significant (*p* < 0.1). Thus, the advantage of the constant mapping of voice and location in the medium-uncertainty condition was more robust in terms of RT than accuracy. For both measures, the ecological validity of the constant mapping in the medium-uncertainty condition led to better performance, equal to that in the low-uncertainty condition, despite having the same degree of stimulus variability across trials as in the high-uncertainty condition.

#### Performance with early and late target words

**Figure 3:**
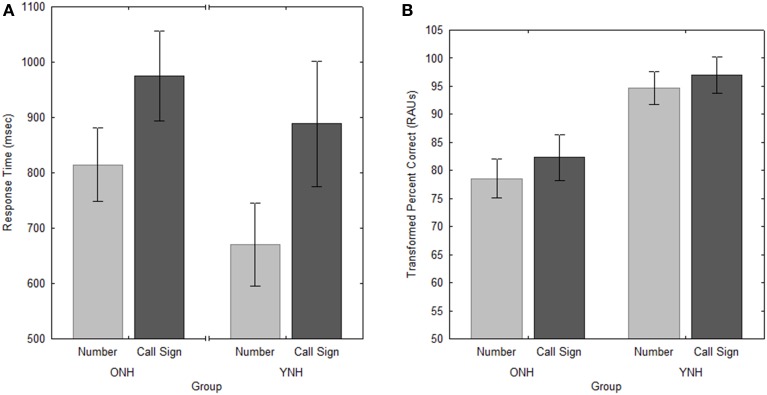
**Response times (A) and accuracy (B) for both groups of subjects in the two Target conditions in Experiment 1**. Error bars indicate ± one standard error.

Figure 3 shows performance as a function of the target word for both groups. It can be seen that both YNH and ONH subjects were consistently slower [*F*_(1, 20)_ = 38.1, *p* < 0.001, $\eta_{\text{p}}^{2}=0.66$], but slightly more accurate [*F*_(1, 20)_ = 7.5, *p* < 0.05, $\eta_{\text{p}}^{2}=0.27$], when responding to the call sign than to the number target. Because the call sign occurred early in each sentence, subjects had more time to prepare their response before the “yes” and “no” response buttons appeared (and the RT timer started) at the end of the sentence presentation. That subjects were unable to use this time to decrease RT suggests that the irrelevant words following the call sign may have interfered with memory or decision processes. The slightly more accurate responding may be due to the greater distinctiveness for call signs (highly distinguishable two-syllable names), compared to numbers, which were more similar single-syllable (with the exception of “seven”) numerals.

### Discussion

In addition to providing measures of working-memory abilities, this modified version of the *n*-back task, using full sentences from the CRM corpus, was designed to assess recall abilities using a listening situation that had some features in common with a natural sequential multitalker conversation. In many ways, performance on this task was similar to that obtained with versions of the *n*-back task that used much simpler visual stimuli and similar strategies for varying *n* (e.g., Verhaeghen and Basak, 2005; Oberauer, 2006; Basak and Verhaeghen, 2011). Both younger and older subjects showed a significant switching cost as evidenced by an increase in RT as *n* (the number back) increased from 1 to 2, and neither group showed any further increases in RT as *n* increased from 2 to 5. It is important to remember that *n* in the present study is not equal to the set size, as is common in *n*-back studies. Because set size is held constant here at 3 (the number of locations), any increase in RT with an increase in *n* would be attributed to an increase in the time between comparison items rather than to an increase in the number of items in a search set. The results also agreed with the earlier visual *n*-back studies in showing no age differences in the switching cost. However, in contrast to the earlier studies, no correction for general slowing was required, because RTs were very similar for younger and older subjects. Thus, not only were there no age differences in accessibility of items in the focus of attention, there was little or no evidence of slowing of memory retrieval or decision making with age in this task.

On the other hand, age differences *were* observed with accuracy in the present task. Older subjects were consistently less accurate, by about 10 percentage points, than younger subjects for all values of *n*. In the related visual *n*-back studies, age differences were not found for *n* = 1 when set size was confounded with the number back, but, when they were not confounded, as in the present experiment, age differences were also found for all values of *n* (Verhaeghen and Basak, 2005; Basak and Verhaeghen, 2011). Thus, older subjects appear to have more trouble maintaining an item in memory, whether it is in the focus of attention or in the outer store, at least under some task conditions. Despite this, when older subjects correctly recalled the repetition of the current item (or lack of it) in a given location, they were not significantly slower than younger subjects in recall and decision making. Thus, aging appears to affect the ability to hold information in memory in this task, but not the ability to access and make judgments on that information when the information is available.

Variability in talkers and nontarget words across trials in this task had a detrimental effect on performance for both younger and older subjects. Subjects were fastest and most accurate when the talker and nontarget words were held constant across trials (low uncertainty) and slowest and least accurate when those words varied randomly (high uncertainty). However, when talkers were assigned to unique locations (as they typically are in most conversational settings), performance was just as good as in the low-uncertainty condition, despite the same amount of talker and semantic variability as in the high-uncertainty condition. This shows that both older and younger listeners are sensitive to location information and voice information, and that a consistent mapping of these two types of information is helpful when trying to keep track of what was said in a sequence of spoken sentences. In a sense, this mapping can be thought of as a reduction of uncertainty, in that subjects know what voice to expect from each location. But because subjects cannot predict which location will be used on the next trial and cannot identify the location or the talker until after the sentence begins, it seems unlikely that the consistent mapping advantage is simply due to reduced variability in the mapping of stimulus properties that are irrelevant to the task. It seems more likely that the variable talker-location mapping adversely affects performance because it is a violation of an expectation based on everyday experience.

The findings with regard to selection of the early-occurring target (call sign) or late-occurring target (number) are difficult to interpret. Both groups were substantially slower in making judgments about the repetition of the call sign, but they were slightly more accurate than with the number target. While this is consistent with a speed-accuracy tradeoff, the significant, but rather small, increase in transformed percent-correct performance (less than 2 RAU) may not entirely account for the relatively large increase in RT (nearly 200 ms, or about 26%) and it is not clear why subjects would use a different speed-accuracy tradeoff based on the target word. Despite giving subjects more time to prepare their response before the sentence ended (and the RT clock started), the greater time and number of intervening words between the early target word and the presentation of the response options appears to have made it more difficult for subjects to access the item in memory. It thus appears that this retroactive interference slowed recall and/or decision processes without affecting the availability of the target word in memory.

## Experiment 2: Older hearing-impaired adults with and without amplification

The older subjects in Experiment 1 generally performed well on the auditory *n*-back task, but although they were about as fast as the younger subjects in all conditions, they were consistently less accurate. This pattern of results suggests that, at least with the present task, aging affects the ability to hold information in working memory while processing new information, but not the ability to access information in working memory and make rapid judgments based on it. However, the older subjects in Experiment 1 had relatively good hearing and showed no evidence of having any difficulty understanding the talkers. Because hearing loss is common in the older population, it is important to determine whether older listeners with poorer hearing perform differently on this type of auditory memory task. If listeners have to expend more effort trying to understand what is being said, they may be more susceptible to memory interference and uncertainty in ways that lead to a different pattern of results from that observed in Experiment 1 (see McCoy et al., 2005; Pichora-Fuller and Singh, 2006; Gosselin and Gagné, 2011; Rudner et al., 2012; Yusuf et al., 2012).

To examine the effect of hearing loss on performance with this task, Experiment 2 employed a group of hearing-impaired subjects who performed the auditory *n*-back task with and without custom spectral shaping (amplification) to ensure audibility of the speech materials. It was expected that without spectral shaping, the added difficulty would cause these listeners to be: (1) slower than their normal-hearing age peers; (2) more affected by stimulus uncertainty; (3) less able to take advantage of location and voice cues, and thus less able to take advantage of a constant talker-location mapping; and (4) more affected by target position because of a greater susceptibility to interference from irrelevant words following the early target. With shaping, these listeners were expected to be more like the older normal-hearing listeners. However, because this group may suffer from cochlear pathology and may have undergone changes in higher-level processing, either central auditory or cognitive processing (Humes et al., 2012), they were not expected to perform the same as the ONH listeners in Experiment 1.

### Methods

#### Subjects

**Figure 4:**
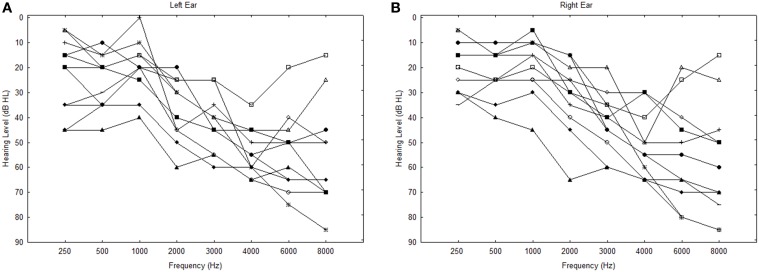
**Hearing thresholds for the left (A) and right (B) ears of the OHI subjects in Experiment 2**.

The subjects in this experiment were 11 older hearing-impaired listeners whose ages ranged from 64 to 85 years (mean = 70.1 years; *SD* = 5.7). There were five females and six males; two were current hearing aid users, and the others had never worn hearing aids. The highest level of education completed ranged from high school (one subject) to vocational school (two subjects), college (four subjects), and graduate school (four subjects). All subjects had symmetrical high-frequency sensorineural hearing loss and failed to meet the definition of normal hearing used in Experiment 1 (as described above). Thresholds for all subjects are shown in Figure 4. Except for hearing thresholds, the inclusion criteria were the same as for the older subjects in Experiment 1, and all had previously participated in the same individual differences study by Humes et al. (2013) as had the ONH subjects in Experiment 1.

#### Stimuli

The stimuli were the same CRM sentences used in Experiment 1, presented with and without custom amplification to ensure audibility. In the unshaped condition, the same 85-dB SPL level used in Experiment 1 was used in this experiment. In the shaped condition, presentation levels were adjusted to ensure that speech information was audible and to provide comparable presentation levels for all listeners. The levels were adjusted by measuring the long-term spectrum of the full set of stimuli and filtering each stimulus to shape the spectrum according to each listener's audiogram. The shaping was applied with a 68 dB SPL overall unshaped speech level as the starting point, and gain was applied as necessary at each 1/3 octave band to produce speech presentation levels at least 13 dB above threshold from 125 Hz to 4000 Hz.

#### Procedures

Testing procedures were the same as in Experiment 1, using the same equipment. All subjects were tested twice: once with shaping and once without shaping, each time following the same procedures and including all the conditions described for Experiment 1. Based on a random assignment, five subjects were tested with unshaped stimuli first, and six were tested with shaped stimuli first. Testing was completed in four 90-min sessions on four separate days.

At the end of the experiment, a short recognition test was conducted to determine whether subjects were able to understand the words in the CRM sentences at the levels used in the experiment. The sentences were presented both with and without shaping, using the right ear only. Subjects listened to the same CRM sentences used in the main experiment (using the same talkers) and indicated the call sign, color, and number in each sentence by touching (or clicking with a mouse) virtual buttons on the monitor labeled with all of the possible options for each of the three target words. There were 16 blocks of 32 trials: 8 blocks with shaping and 8 blocks without shaping, using the same counterbalanced order of shaping conditions used in the main experiment.

### Results

On the post-experiment recognition test, all subjects correctly identified all target words on every trial, clearly demonstrating that the stimuli were audible under the presentation conditions used in this experiment. Thus, the deviations from perfect performance described below must be attributed to the memory and processing requirements of the task.

Response times and accuracy were analyzed as in Experiment 1, using the same exclusion criteria for outliers in the RT data and resulting in similar rejection rates. Overall, subjects' accuracy was very close to the 78% correct obtained with the ONH subjects in Experiment 1, with 80% correct overall for both shaped and unshaped testing. However, RTs were considerably slower. Average RTs across all conditions were 1561 ms (*SD* = 544 ms) without shaping and 1475 ms (*SD* = 618 ms) with shaping, a nearly 70% increase relative to the ONH subjects in Experiment 1. The slow mean response time for this older group was partly due to one listener (the oldest, at 85 years) whose average RT was about 2.6 standard deviations above the group mean. (This subject was retained because performance was above chance and response times showed systematic variation with conditions.) However, even without this subject, mean performance was still 470 ms slower than for the ONH subjects in Experiment 1. This difference was statistically significant whether evaluated with or without the slowest subject [*t*_(20)_ = 3.26, *p* < 0.005 and *t*_(19)_ = 3.79, *p* < 0.005, respectively].

Analysis of variance was performed, using a 2 (shaping/no shaping) by 3 (Uncertainty) × 2 (Target) × 5 (*n*-back) design for both RT and percent-correct performance (RAU transformed). No effect of shaping was observed for either RT or tPC (*F*s < 1.0), and there were there no interactions with shaping for either measure (*p* > 0.05). As in Experiment 1, there was a significant effect of *n* for both RT [*F*_(4, 40)_ = 7.74, *p* < 0.001, $\eta_{\text{p}}^{2}=0.44$] and tPC [*F*_(4, 40)_ = 53.60, *p* < 0.001, $\eta_{\text{p}}^{2}=0.84$], as well as significant effects of Target [for RT, *F*_(1,10)_ = 7.60, *p* < 0.05, $\eta_{\text{p}}^{2}=0.43$; for tPC, *F*_(1,10)_ = 16.63, *p* < 0.005, $\eta_{\text{p}}^{2}=0.62$], but the Uncertainty manipulation did not have a significant effect in this Experiment (*p* > 0.05 for both RT and PC).

**Figure 5:**
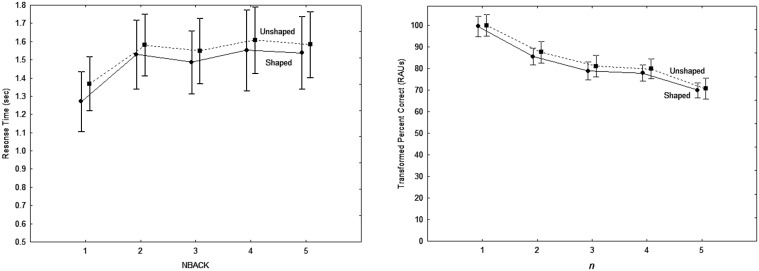
**Response times for correct responses (A) and accuracy (B) as a function of the number of presentations separating the target words to be compared, for OHI subjects with and without shaping in Experiment 2**. Error bars indicate ± one standard error.

The main results are summarized in Figure 5, which shows RT as a function of the number back (*n*) in Figure 5A, and tPC vs. *n* in Figure 5B. The pattern of performance for both RT and tPC was essentially the same as in Experiment 1. There was a clear cost of switching information in and out of the focus of attention, as seen by the increase in RT between *n* = 1 and *n* = 2 (Tukey HSD, *p* < 0.01), with no significant changes in RT with further increases in *n* (*p* > 0.05). Also as in Experiment 1, the decrease in tPC with *n* was significant for successive increases in *n* (Tukey HSD, *p* < 0.01), except for that between *n* = 3 and *n* = 4 (*p* > 0.05).

A significant Uncertainty by *n*-back interaction [*F*_(8, 80)_ = 2.8, *p* < 0.01, $\eta_{\text{p}}^{2}=0.22$] in the RT data was primarily due to a reduced switching cost for the high-uncertainty condition. This was the only Uncertainty condition in which performance was not consistently better for *n* = 1 than for *n* > 1, with RT for *n* = 1 not significantly better than for *n* = 3 or *n* = 5 (Tukey HSD, *p* > 0.05). A significant 3-way interaction between Target, Uncertainty, and *n*-back [*F*_(8, 80)_ = 3.3, *p* < 0.01, $\eta_{\text{p}}^{2}=0.25$] reflected the fact that this reduced switching cost was greater for the number than for the call-sign target.

There were two significant interactions in the tPC data. A Target by *n*-back interaction [*F*_(4, 40)_ = 6.7, *p* < 0.001, $\eta_{\text{p}}^{2}=0.40$] was due to the lack of a significant effect of Target for *n* = 1 or for *n* = 5 (Tukey HSD, *p* > 0.5). An Uncertainty by *n*-back interaction [*F*_(8, 80)_ = 2.3, *p* < 0.05, $\eta_{\text{p}}^{2}=0.19$] reflected a tendency for greater differences between the three uncertainty conditions for *n* = 1 and *n* = 5 than for other values of *n*.

**Figure 6:**
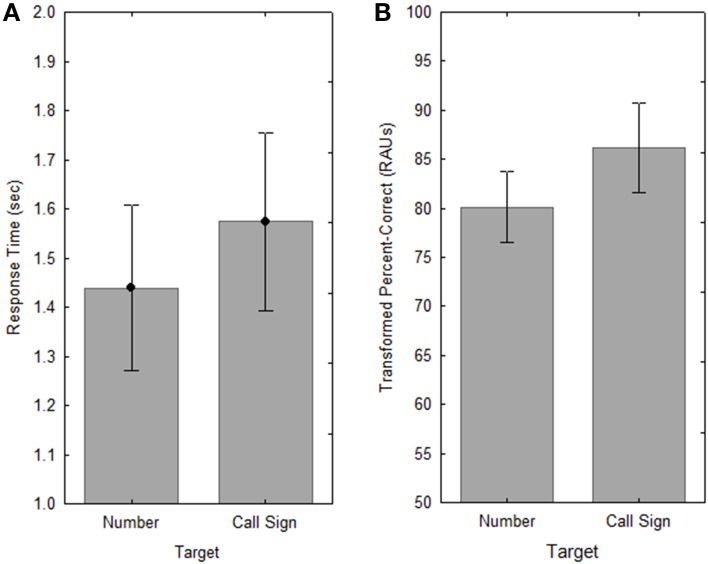
**Response times (A) and accuracy (B) for the two Target conditions in Experiment 2**. Error bars indicate ± one standard error.

The effect of Target was essentially the same as in Experiment 1, with significantly slower RT [*F*_(1, 10)_ = 7.6, *p* < 0.05, $\eta_{\text{p}}^{2}=0.43$] and greater accuracy [*F*_(1, 10)_ = 16.6, *p* < 0.005, $\eta_{\text{p}}^{2}=0.62$] for judgments of repetition of the call sign in a given location than for repetitions of the number (see Figure 6). This is suggestive of the same speed-accuracy tradeoff seen in Experiment 1, although the increase in RT for the call sign was slightly smaller than that seen with the older listeners in Experiment 1 (approximately 135 ms; a 9% increase) and the corresponding change in PC of roughly 4 percentage points was slightly higher.

**Figure 7:**
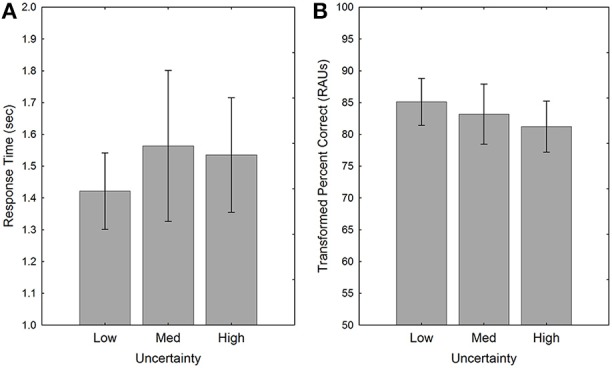
**Response times (A) and accuracy (B) for the three Uncertainty conditions in Experiment 2**. Error bars indicate ± one standard error.

Although the effects of target identity (or sentence position) and the number of intervening sentences between to-be-compared items were quite similar to those observed in Experiment 1, this was not the case with the uncertainty manipulation. Although there was a slight tendency for RT to increase and for accuracy to decrease as the level of uncertainty increased (see Figure 7), these differences were not significant, and there was no evidence of an advantage for the consistent mapping of talker and location, as observed in Experiment 1.

## General discussion

These experiments used a modified *n*-back task with auditory presentation of sentences to examine the effects of aging and hearing loss on the ability to understand and remember spoken material and to keep track of source locations. By asking subjects to compare a target word in a sentence just heard to the corresponding target word in the last sentence presented from the same location (left, center, or right), the task eliminates the need to keep track of the number of trials between comparison items, as is commonly required with the *n*-back task. This makes the task more natural, and when a specific talker is associated with a specific location, the task becomes similar to keeping track of who said what in a typical conversational setting.

In many respects, the pattern of results was similar to that from earlier studies using visual presentation of digits in which the to-be-compared items were also identified by location (a column in a visual display), rather than by a fixed number back in a series of presentations (e.g., Verhaeghen and Basak, 2005; Basak and Verhaeghen, 2011). The added complexity of full sentences, rather than single digits, did not change the basic pattern of response times and accuracy as a function of the number back (*n*). There was a similar cost of switching items in and out of the focus of attention, as evidenced by the increase in RT between *n* = 1 and *n* = 2, and no further increases in RT as *n* increased beyond 2. However, unlike the earlier visual studies, the set size was held constant, and changes in *n* were associated only with longer delays between items to be compared. In the earlier studies, set size was varied and results were plotted as a function of set size whether it was confounded with the number back (as in Verhaeghen and Basak, 2005) or varied independently from the number back (as in Oberauer, 2006; Basak and Verhaeghen, 2011). Basak and Verhaeghen did not examine the effect of the number back, partly because the focus was on set size (which was equal to the number of positions), but also because of the constraint that all positions must be tested before any position is repeated. With this constraint, the position to be tested on a given trial becomes more predictable as the number of untested positions in the set decreases. However, Oberauer (2006) did not use the same position-sampling constraint and could thus examine the effect of the number back (or *lag*) independently of set size. He found that both RT and PC were affected by lag as well as by set size, with linear increases in RT and linear decreases in accuracy as the lag increased. There was no evidence of the flattening of the RT function after *n* = 2, as observed in the present study.

Given the use of sentences in the present study, which introduce longer presentation times (and intertrial intervals) and a greater potential for interference than single digits, it is perhaps surprising that response times did not increase when the number of intervening sentences between compared target words increased. However, accuracy did decrease linearly with *n*, suggesting that memory interference and decay were occurring with time and number of stimuli presented. The lack of an effect of the number of intervening sentences on response times for correct responses after *n* = 2 (when a focus switch was required) indicates that when the information is available in memory, access time and decision time are not slowed. Thus, it is primarily the likelihood of a correct response (or the availability of information) that decreases with *n*, not the accessibility of the information stored in memory.

The present findings provide no evidence to suggest that the effect of the number back on response times or accuracy changes with age or hearing loss. Although the older hearing-impaired listeners in Experiment 2 were substantially slower than those in Experiment 1, they showed roughly the same switching cost and no further increases in response times with increasing values of *n*. Moreover, there was no main effect of age on response times in Experiment 1 and there were no interactions involving age. The only effect of age was on accuracy, but there were no interactions involving age for the accuracy measure either. Older subjects were less accurate than younger ones, but this did not vary with the number back or any other experimental manipulation in Experiment 1. Thus, it appears that aging primarily affects the susceptibility to decay and interference of information inside and outside of the focus of attention, while having little or no effect on the accessibility of information that is retained.

Age differences were also absent in the effect of target word location. It was expected that older subjects might have more trouble with the early (call sign) targets because of a greater susceptibility to interference from the following words in the sentence. In this task, a judgment about the repetition of a target word can be made as soon as the word is recognized, but the response cannot be made until the end of the sentence, when the response options are presented (and the response timer starts). Thus, faster responding would be expected for early targets if subjects could make their judgments early and prepare their response while ignoring the rest of the sentence. However, both younger and older subjects were unable to take advantage of this, responding more slowly to early targets than to later (number) targets, despite being slightly more accurate with the early targets. Although it is not clear why responses were slower for the early targets, both groups appear to have required more time for recall and decisions regarding the early targets, even though the information was at least as available (as indicated by accuracy scores) as it was for the later targets. It may be that interference or distraction from words following the target word make younger and older listeners less confident in their responses, thus slowing response times without affecting accuracy.

Although the OHI subjects in Experiment 2 showed the same effect of *n* and target word on response times and accuracy, they had much slower response times than the ONH subjects in Experiment 1, with a mean difference of more than 600 ms (roughly 1.7 times greater). The difference was fairly consistent across subjects; only three subjects in Experiment 2 had mean RTs below 1 sec, while all but 3 of the 11 ONH subjects in Experiment 1 had RTs below 1 sec. Shaping, to ensure audibility, had no effect on response times or accuracy, and none of the effects in Experiment 2 were impacted by the shaping manipulation. The slower response times do not appear to be due to an inability to reliably understand the target words, because subjects were as accurate as the ONH subjects in Experiment 1 even without shaping, and they performed perfectly on a target-word recognition test using the same stimulus materials presented at the same levels used in Experiment 2. Although the average age for the OHI group was about 5 years greater than that for the ONH group, age was not significantly correlated with RT. The oldest subject (85 years) was the slowest by a large margin, but, with this extreme subject excluded, the correlation between age and RT for all HI subjects was 0.05. Finally, even cognitive abilities, as measured by a global cognitive ability factor obtained in an earlier study (Humes et al., 2013), do not account for the slower response times. The extremely slow subject did score quite poorly on the cognitive measure (based on three working memory measures and a processing speed test), but the correlation between that measure and RT was small and non-significant, with (*r* = −0.26) or without (*r* = 0.14) the extreme subject included.

Another potential explanation for the substantially slower RTs in Experiment 2 is that these hearing-impaired subjects had to expend more effort to understand the spoken sentences than did the normal hearing listeners in Experiment 1. Subjects with mild to moderate hearing loss often must expend more effort than their normal-hearing peers to achieve comparable levels of speech understanding, and this is not always evident in speech-recognition performance (see Rabbitt, 1991; Pichora-Fuller et al., 1995; Tun and Wingfield, 1999; McCoy et al., 2005; Gosselin and Gagné, 2011). This research suggests that the emphasis on memory over immediate recognition in the current task would be expected to make it sensitive to differences in effort, especially in an older population, which is likely to have declining cognitive abilities. The shaping used in Experiment 2 should have reduced the amount of effort required by reducing reliance on partial information to compensate for inaudible portions of speech. However, the lack of an effect of shaping does not rule out an effort-based explanation for the slower response times in Experiment 2. Although the provided shaping ensures audibility from 125 to 4000 Hz, the listening experience is not equivalent to that for normal-hearing listeners. The listeners in Experiment 2 were not experienced hearing aid users (only two had ever worn hearing aids), and the amplified speech signal presented cannot be expected to provide a listening experience equivalent to normal hearing. Support for the effort explanation was also lacking in the correlations between hearing loss (PTA and HFPTA) and RT within this group of OHI listeners: the correlations were not significant and the tendency was in the wrong direction (with greater hearing loss associated with slightly faster response times). However, lack of an association between hearing loss and RT within a small group of hearing-impaired listeners is not strong evidence against the effort explanation.

It thus appears that the considerably slower response times of the OHI group may be due to an increase in the effort required to understand speech, which is commonly associated with hearing loss. That accuracy was essentially the same as for the ONH subjects in Experiment 1 indicates that the OHI subjects understood and retained the target words about as well as the ONH subjects. The longer response times thus indicate difficulty accessing the stored information, lower confidence in their judgments, or both. Although lower confidence is often associated with longer response times (e.g., Emmerich et al., 1972; Vickers and Packer, 1982), it is not possible to determine the relative contributions of access time and decision time to response latencies in the present study.

### Uncertainty and the use of location and voice information

The use of different talkers and virtual spatial locations in this study allowed for an examination of the ability to use location and voice information in a speech-understanding task as a function of age and hearing loss. It also allowed for the introduction of greater stimulus variability across trials by varying location and talker as well as the words (target and nontarget) used in the CRM sentences. The Uncertainty variable in this study included three levels of stimulus variability, or uncertainty, that utilized two types of assignment of talkers to spatial locations: consistent and variable. The normal-hearing subjects in both age groups in Experiment 1 were affected the same way by the uncertainty manipulation. Responses were slower and less accurate with the highest level of uncertainty, when the voice, location, and nontarget words varied randomly over trials, than in the low-uncertainty condition, in which the same voice and nontarget words were used on every trial. However, in the medium-uncertainty condition, with consistent mapping of talkers to locations (but with the same amount of stimulus variability as the high-uncertainty condition), response times were roughly the same as in the minimal-uncertainty condition, and accuracy followed a similar pattern. Thus, the decline in performance across the three uncertainty conditions was almost entirely due to the difference between consistent and inconsistent mapping of voice and location. Although the use of a single talker in three locations in the minimal uncertainty condition is not a natural situation, this was offset by the lack of variability in voice and nontarget words. When there was variation in talkers (voices), the ecological validity of a consistent location for each talker eliminated the effect of the increased stimulus variability on response times and nearly so for accuracy. This suggests that it was the unpredictable change in talkers (not simply variation in the talker and the nontarget words) that was primarily responsible for the increased difficulty in the high-uncertainty condition.

In contrast to the normal-hearing listeners, the older hearing-impaired listeners in Experiment 2 were unable to take advantage of the consistent voice/location mapping in the medium-uncertainty condition. Although there were small differences between uncertainty conditions favoring minimal uncertainty, the effect of uncertainty was not significant in this group, and response times with consistent mapping were nearly identical to those with the inconsistent mapping of the high-uncertainty condition. Thus, despite being just as good in recognition and recall accuracy as the ONH subjects in Experiment 1, the OHI subjects did not find the predictability of a consistent mapping of voice and location information helpful. Given that the ability to discriminate the three virtual locations is required to perform this task, it is unlikely that localization problems were a significant factor. However, difficulty in reliable discrimination of the three voices may have been responsible for the failure to benefit from consistent mapping. Although the three talkers used in this study are highly discriminable for young normal-hearing listeners, the older hearing-impaired listeners may not have been as sensitive to the voice differences. However, given that older hearing-impaired listeners have been shown to be adversely affected by talker uncertainty in recognition tasks using these CRM stimuli (Humes et al., 2006; Humes and Coughlin, 2009), it is unlikely that poor talker discrimination abilities fully account for the lack of benefit in the consistent mapping condition. It seems more likely that the same factors that cause OHI listeners to require more time to make memory-based judgments also reduce their sensitivity to more subtle stimulus characteristics that are not a necessary component of the task. That is, the reduction in available resources, due to the increased effort expended by OHI listeners when listening to the sentences, may also reduce their sensitivity to talker differences in the context of a multitalker listening task that emphasizes memory over recognition abilities.

## Summary and conclusions

This study used a modified auditory *n*-back task with multiple talkers and locations to approximate the demands of a sequential multitalker conversation. Young and older adult listeners, with and without hearing loss, were asked to judge whether a target word in a sentence just heard was the same as in the last sentence heard from a given location. Performance on this task was similar to that obtained in a comparable version of the *n*-back task in the visual modality. Younger and older subjects with normal hearing showed similar costs when switching information in and out of the focus of attention and had similar response times overall. Neither group showed any increase in response times with greater numbers of trials between comparison words when comparing target words outside of the focus of attention (i.e., for comparisons with words presented more than one trial back in the sequence). Age did have an effect on the accuracy of the judgments; both groups were less accurate as the interval between comparison words increased, but older subjects performed consistently worse for all intervals between comparison words, whether or not focus switching was required. Older subjects with hearing loss showed a similar pattern of results, but had considerably longer response times, despite responding as accurately as the older normal-hearing listeners. All subjects responded more slowly and slightly more accurately to early target words than to later target words, showing no evidence of differential interference with age or hearing loss from the greater number of irrelevant words following the early target word.

Normal-hearing listeners in both age groups showed essentially the same adverse effect of stimulus uncertainty, but performed better under high uncertainty when talkers were consistently assigned to specific locations, rather than varying randomly across trials. However, older hearing-impaired listeners, in addition to responding more slowly than older normal-hearing listeners, showed no effect of stimulus uncertainty and were not helped by the ecological validity of a consistent mapping of voice and location. The slower response times and insensitivity to consistent talker/location mapping for the older hearing-impaired listeners, despite accuracy equal to that for the normal-hearing older adults, suggest that the older hearing-impaired listeners may have exerted more effort to perform at the same level of accuracy. This may have led to slower response times (perhaps related to reduced confidence) and reduced sensitivity to voice characteristics that can be helpful in reducing talker uncertainty (when talker identity is predicted by the location) and facilitating target word comparisons when the words are spoken by the same talker. It should be noted that these effort-based effects were observed using presentation levels well above typical conversational levels, and with customized spectral shaping. It is likely these effects would have been greater if the stimuli were presented at normal conversational levels.

These findings show that when simple speech-recognition tasks are complicated by memory requirements that begin to resemble the demands of a typical sequential multitalker conversation, hearing impairment, especially when combined with aging, can make it more difficult to keep track of what has been said and by whom. Although hearing loss primarily affected response times rather than accuracy in the present study, slower response times may result from greater effort, which can cause fatigue and reduce accuracy after prolonged periods of listening, especially under more difficult listening conditions. Moreover, a reduction in attentional resources that results in reduced sensitivity to voice characteristics may also diminish a listener's ability to notice other indexical properties or prosodic information that can be critical for effective communication.

## Conflict of interest statement

The authors declare that the research was conducted in the absence of any commercial or financial relationships that could be construed as a potential conflict of interest.
